# Antibiotic-resistant profile and the factors affecting the intravenous antibiotic treatment course of generalized Staphylococcal Scalded Skin Syndrome: a retrospective study

**DOI:** 10.1186/s13052-021-01120-6

**Published:** 2021-08-06

**Authors:** Tao Yang, Jiangyi Wang, Junya Cao, Xinyue Zhang, Yun Lai, Longnian Li, Xiaoying Ye, Cong You

**Affiliations:** 1 Department of Dermatology and Venereology, Candidate Branch of National Clinical Research Centre for Skin and Immune Diseases, The First Affiliated Hospital of Gannan Medical University, No. 23 Qingnian Road, Zhanggong District, Ganzhou, 341000 China; 2 Department of Dermatology and Venereology, The General Hospital of Tianjin Medical University, No. 154 Anshan Road, Heping District, Tianjin, 300052 China

**Keywords:** Antibiotic sensitivity, Intravenous antibiotic treatment, Staphylococcal Scalded Skin Syndrome, *S. aureus*

## Abstract

**Background:**

Staphylococcal Scalded Skin Syndrome (SSSS) is caused by a special type of *Staphylococcus aureus (S.aureus)* which can produce exfoliative toxins. The generalized SSSS is recommended to be admitted and treated with intravenous antibiotics. However, there were limited reports on whether personal and clinical factors can have impacts on the duration of intravenous antibiotic application for pediatric patients with generalized SSSS.

We performed a study to assess the factors affecting intravenous antibiotic treatment course of SSSS patients. Additionally, the positive culture rates of *S.aureus* in different samples and the antibiotic-resistant profile were investigated.

**Methods:**

Two hundred nineteen patients with generalized SSSS were included. Gender, age, area, season, maximum axillary temperature, white blood cell (WBC) count, C-reactive protein (CRP) level, types of intravenous antibiotics, and types of external antibiotics were recorded as the baseline. Simple linear regression was applied in the univariate analysis to determine the variables with statistical significance and then these variables were further examined in multivariate linear regression model. The positive culture rates of *S.aureus in* different sample sources were calculated and the drug sensitivity results were statistically compared by pairwise Chi square test.

**Results:**

According to the multiple linear regression, older ages (β = − 0.01, *p* < 0.05) and external application of fusidic acid (β = − 1.57, *p* < 0.05) were associated with shorter treatment course, elevated leukocytes (β = 0.11, *p* < 0.001) and CRP level (β = 1.64, *p* < 0.01) were associated with longer treatment course. The positive culture rates of periorificial swabs, throat swabs, and blood samples were 54.55, 30.77, and 5.97% respectively. The resistant rates of levofloxacin (8.33%), gentamycin (8.33%), tetracycline (25%), oxacillin (8.33%), vancomycin (0%) were significantly lower than the ones of erythromycin (100%), trimethoprim-sulfamethoxazole (TMP/SMX) (83.33%), clindamycin (91.67%), penicillin G(100%) (*p* < 0.001).

**Conclusion:**

Elevated leukocytes and CRP level indicated prolonged intravenous antibiotic treatment course. Older ages and external application of fusidic acid helped to reduce the treatment course. Compared with blood samples, the culture positive rates of *S.aureus* in periorificial and throat swabs were higher. Oxacillin and vancomycin resistance was rare and clindamycin resistance was common. Clindamycin monotherapy for SSSS should be avoided.

## Background

Staphylococcal Scalded Skin Syndrome (SSSS) is caused by a special type of *(S.aureus)* which can produce exfoliative toxins. There are two forms of SSSS including the generalized SSSS and the localized one which is also called bullous impetigo. Erythematous blisters and honey-colored crusts especially in the diaper area were found in bullous impetigo. Most of the body surface is involved in generalized SSSS, which may lead to critical condition of the patients [1, 2]. The exfoliative toxin A (ETA) and exfoliative toxin B (ETB) are responsible for the detachment of the granular layer of the skin in patients with generalized SSSS [3]. The neonates and children below 5 years old are predisposed to be affected by SSSS [1, 4, 5]. Recently, an upward trend of morbidity of SSSS was observed despite the low rate of occurrence of SSSS [6]. According to an epidemiological study, the overall incidence of SSSS increased by 47.1% during 2010 and 2014, and the number of admissions for SSSS during the period reached 6143 in the United States. It is worth noting that there were rapid increase of annual morbidity among children below 17 years old [7]. Higher incidence was found in developing countries where the incidence of staphylococcal infections is higher [8].

Generalized SSSS may cause urgent admissions to hospital wards, mainly due to the severe tenderness of the children’s skin and the potentially fatal severe complications such as sepsis. Prolonged duration of intravenous antibiotic application generates long hospital stay, significant costs and are a significant financial burden for the health care system. However, there were limited reports on whether personal and clinical factors can have impacts on the duration of intravenous antibiotic application for pediatric patients with generalized SSSS. The aim of the study reported here was to assess the factors affecting the duration of intravenous antibiotic application of patients admitted to hospital due to generalized SSSS. Additionally, the positive culture rates of *S.aureus* were calculated in different sample sources and the drug sensitivity results were compared in different antibiotic groups.

## Materials and methods

### Patients

The study was based on a retrospective analysis of clinical data of patients referred to hospital due to generalized SSSS and subsequently admitted to the Department of Dermatology, The First Affiliated Hospital of Gannan Medical University between January 2012 and June 2020. Patients potentially eligible for inclusion in the study were pre-identified by searching the electronic database of the hospital, using the International Classification of Diseases, tenth revision (ICD-10) codes L00.× 00 (for SSSS) and L01.000 × 013 (for impetigo). The inclusion criteria of our study were those patients who were admitted into the Department of Dermatology without any complications and comorbidities. As the diagnosis of SSSS is mainly clinical, we applied the items “tender erythroderma, bullae, and desquamation with a scalded appearance and flexural accentuation, characteristic periorificial (e.g. perioral, periocular) crusting and radial fissuring, positive Nikolsky sign, and absence of mucosal involvement” as diagnostic criteria for SSSS [9, 10]. In our study, we aimed to assess the factors affecting the intravenous antibiotic treatment course, so the patients with generalized SSSS were included, localized form of SSSS were excluded because most of them were treated with oral antibiotics at home. Exclusion criteria were as follows: patients whose caregivers refused the application of intravenous antibiotics; patients with comorbidities such as urinary tract infection, bronchitis, or pneumonia due to bacterial infection which may have an effect on the duration of intravenous antibiotics; patients with complications such as sepsis and severe dehydration; patients with severe immunocompromised diseases such as congenital immune deficiency or impaired kidney function. The diagnosis of pediatric sepsis was made according to the criteria established by International Pediatric Sepsis Consensus Conference. The definition of sepsis is that Systemic Inflammatory Response Syndrome (SIRS) in the presence of or as a result of suspected or proven infection [11] .

**Fig. 1:**
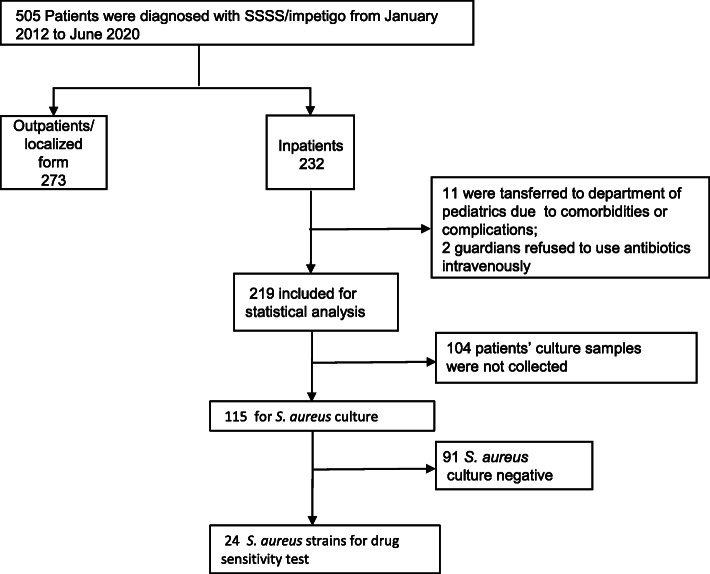
Study flowchart of materials and methods. SSSS: Staphylococcal Scalded Skin Syndrome; *Staphylococcus aureus: S. aureus*

A total of 505 patients were identified, 273 patients were excluded from the study because they were diagnosed with the localized form of SSSS or treated as outpatients. Among the 232 inpatients, 11 patients were transferred to the pediatric department due to severe complications of sepsis or comorbidities such as pneumonia or bronchitis. Two patients’ caregivers refused the intravenous antibiotic regimen. Finally, 219 patients were eligible to be included in the analysis based on an evaluation of the medical documentation, physical examination, microbiological examination, and laboratory tests (Fig. 1), 115 samples were found to be collected from patients for *S.aureus* culture and 24 *S.aureus* strains were successfully cultured for antibiotic sensitivity test. The baseline characteristics of the patients were collected including gender, age, area, season, maximum axillary temperature, white blood cell (WBC) count, C-reactive protein (CRP) level, types of intravenous antibiotics, and types of external antibiotics. The normal range of CRP level was 0-6 mg/L. The baseline characteristics of the generalized SSSS patients are presented in Table 1.

**Table 1:** Baseline characteristic of patients with generalized Staphylococcal Scalded Skin Syndrome

Characteristics	Cases n(n%)/mean ± SD
Gender	
Male	124 (56.62)
Female	95 (43.38)
Age (months)	25.91 ± 17.40
Area	
Rural	149 (68.04)
Urban	70 (31.96)
Season	
Spring	26 (11.87)
Summer	76 (34.70)
Autumn	76 (34.70)
Winter	41 (18.72)
T_max_(°C)	37.83 ± 0.78
WBC count(×10^9^/L)	11.55 ± 4.28
CRP level	
Normal	210 (95.89)
Elevated	9 (4.11)
Intravenous antibiotic types	
Amoxicillin–clavulanic acid	32 (14.61)
Cefazolin	117 (53.42)
Cefuroxim	70 (31.96)
External antibiotic types	
None	7 (3.20)
2% Mupirocin ointment	196 (89.50)
2% Fusidic acid cream	16 (7.31)

*T*_*max*_ maximum axillary temperature, *WBC* white blood cell, *CRP* C-reactive protein, *SD* standard deviation

### Laboratory tests and treatment regimens

After we searched the electronic database of medical records, culture and drug sensitivity results of 115 samples including 67 blood, 22 periorificial and 26 throat samples were identified. *S.aureus* drug sensitivity tests were performed using the VITEK 2 Compact automatic analysis system (Bio Mérieux, Lyon, France). Three kinds of treatment regimens were found in the prescriptions including amoxicillin–clavulanic acid (Huabei Pharmaceutical Co, Ltd. Shijiazhuang, China) or cefazolin (Huarun Jiuxin Pharmaceutical Co, Ltd. Shenzhen, China) or cefuroxime (Esseti Farmaceutici S.r.l., S. Giorgio a Cremano, Italy), all of the antibiotics above were given intravenously. No antibiotic or 2% mupirocin ointment (China-USA SmithKline Pharmaceutical Co, Ltd. Tianjin, China) or 2% fusidic acid cream (Bright Future Pharmaceutical Co, Ltd. Hongkong, China) was found to be applied externally on the skin lesions twice a day until the withdrawal of intravenous antibiotics.

### Criteria for withdrawal of intravenous antibiotics

According to the medical records, all the patients were fully recovered. The intravenous antibiotics were considered to be withdrawn based on the following: all the erythema gradually resolved without fever, all bullae dried and a few desquamation left with mild pigmentation and no scarring.

### Statistical analysis

SPSS v.23.0 (IBM Corp, Armonk, NY, USA) was used for statistical analysis. Categorized variables were expressed as frequencies, continuous variables were expressed as mean ± standard deviations (SD) or interquartile range (IQR). Mann-Whitney U test or Kruskal-Wallis test were used to compare the differences of antibiotic treatment course among different groups of categorically independent variables for single factor analysis. For the groups of continuously independent variables, simple linear regression was used. Significant confounders (*p* < 0.10) were included in the multiple linear regression model. The differences of sensitive rates of nine antibiotics were compared using Chi square tests. Statistical significance was defined having *p*-value less than 0.05. GraphPad Prism 6.0(GraphPad Software, La Jolla, CA, USA) was used for graphing data.

## Results

Two hundred nineteen patients (95 female and 124 male) were retrospectively enrolled in the study from January 2012 to June 2020, with most of the patients below 5 years old (94.98%). 68.04% patients were from the rural area, 69.40% cases were diagnosed in summer and autumn. The average duration of intravenous antibiotic application was 7.21 ± 1.75 days. All patients achieved full recovery. The CRP levels of the most patients were normal. There were 32, 117, 70 patients treated with amoxicillin-clavulanic acid, cefazolin, or cefuroxime respectively. Seven patients were not treated with external antibiotic, 196 patients were treated with 2% mupirocin ointment and 16 patients were treated with 2% fusidic acid cream. Detailed characteristics of the study subjects are presented in Table 1. The features of the subjects were in line with typical characteristics of SSSS patients.

According to the Mann-Whitney U test or Kruskal-Wallis test for single factor analysis, days of intravenous antibiotic application were different in groups of different CRP levels and external antibiotic applications(*p* < 0.10) (Table 2).

**Table 2:** Single factor analysis of variables affecting the intravenous antibiotic treatment course of generalized SSSS

Characteristics	Group	Days of IV antibiotic treatment (IQR)	*p* value
Gender	Male	(6,8)	0.32
	Female	(6,8)	
Area	Rural	(6,8)	0.52
	Urban	(6,8)	
Season	Spring	(6,8)	0.81
	Summer	(6,8)	
	Autumn	(6,8)	
	Winter	(6,8)	
CRP level	Normal	(6,8)	0.09
	Elevated	(6.5,11)	
IV antibiotic types	Amoxicillin–clavulanic acid	(6,7.75)	0.46
	Cefazolin	(6,8)	
	Cefuroxim	(6.75,8)	
External antibiotic application	None	(7,10)	0.07
	2% Mupirocin ointment	(6,8)	
	2% Fusidic acid cream	(5,7)	

*SSSS* Staphylococcal Scalded Skin Syndrome, *CRP* C-reactive protein, *IQR* (interquartile range), *IV* intravenous

**Fig. 2:**
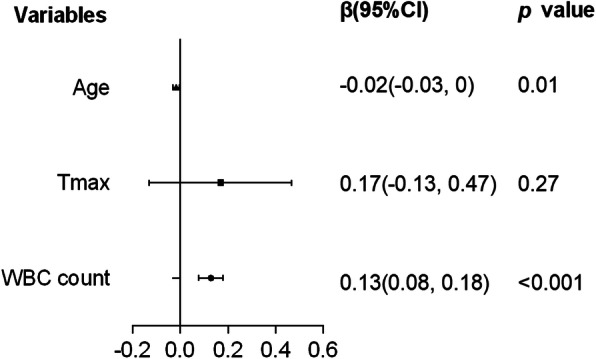
Simple linear regression analysis of variables affecting the intravenous antibiotic treatment course of generalized SSSS. SSSS: Staphylococcal Scalded Skin Syndrome; T_max_: maximum axillary temperature; WBC: white blood cell; CRP: C-reactive protein

**Fig. 3:**
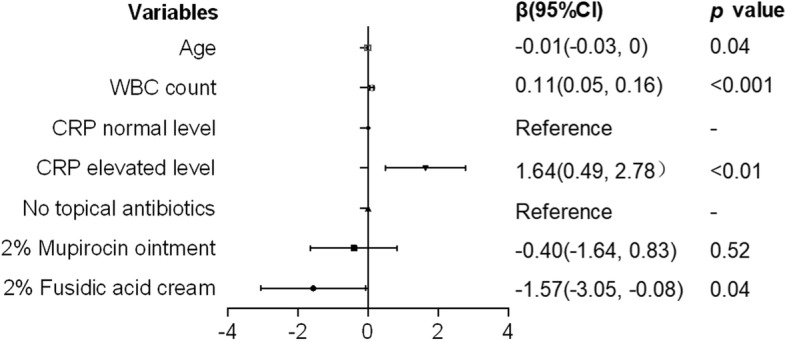
Multiple linear regression analysis of factors affecting the intravenous antibiotic treatment course of generalized SSSS. SSSS: Staphylococcal Scalded Skin Syndrome; WBC: white blood cell; CRP: C-reactive protein

According to the simple linear regression analysis, age (β = −0.02, [95%CI:-0.03,0], *p* < 0.05) and WBC (β = 0.13, [95%CI:0.08,0.18], *p* < 0.001) count were significantly associated with days of intravenous antibiotic application (Fig. 2). In the multiple linear regression model, the associations between the days of intravenous antibiotic application and age (β = − 0.01,[95%CI:-0.03,0], *p* < 0.05), WBC count (β = 0.11,[95%CI:0.05,0.16], *p* < 0.001), CRP elevated level (β = 1.64,[95%CI:0.49,2.78], *p* < 0.01) and external application of 2% fusidic acid cream (β = − 1.57,[95%CI:-3.05,-0.08], *p* < 0.05) remained significant, but the association with external application of 2% mupirocin ointment was not (β = − 0.40,[95%CI:-1.64,0.83], *p* > 0.05) (Fig. 3).

The *S.aureus* culture results of different sample source were presented in Table 3. Twenty four *S.aureus* strains were successfully cultured and the positive culture rates of periorificial swabs, throat swabs, and blood samples were 54.55, 30.77, and 5.97% respectively.

**Table 3:** *S. aureus* detection rates in different sample sources

Sample source	Positive cases n(n%)	Negative cases n(n%)
Periorificial	12 (54.55)	10 (45.45)
Blood	4 (5.97)	63 (94.03)
Throat	8 (30.77)	18 (69.23)

Antibiotic sensitivity results in generalized SSSS-associated *S.aureus* isolates were presented in Table 4.There were significant differences of sensitive rates among nine antibiotics including levofloxacin, erythromycin, gentamycin, tetracycline, Trimethoprim Sulfamethoxazole (TMP/SMX), clindamycin, oxacillin, penicillin G and vancomycin. The pairwise comparison of Chi-square test showed that the resistant rates of levofloxacin (8.33%), gentamycin (8.33%), tetracycline (25%), oxacillin (8.33%), vancomycin (0%) were significantly lower than the ones of erythromycin (100%), TMP/SMX (83.33%), clindamycin (91.67%), and penicillin G(100%) (*χ*^2^ =155.15, *p* < 0.001).

**Table 4:** Antibiotic resistance in generalized Staphylococcal Scalded Skin Syndrome-associated *S. aureus* isolates

Antibiotics	Sensitive n(n%)	Resistant n(n%)	*χ*^2^	p*
Levofloxacin	22 (91.67)	2 (8.33)	155.15	< 0.001
Erythromycin	0 (0)	24 (100)		
Gentamycin	22 (91.67)	2 (8.33)		
Tetracycline	18 (75.00)	6 (25.00)		
TMP/SMX	4 (16.67)	20 (83.33)		
Clindamycin	2 (8.33)	22 (91.67)		
Oxacillin	22 (91.67)	2 (8.33)		
Penicillin G	0 (0)	24 (100)		
Vancomycin	24 (100)	0 (0)		

*TMP/SMX* Trimethoprim Sulfamethoxazole,* Pairwise Chi square test comparison showed that the resistant rates of levofloxacin (8.33%), gentamycin (8.33%), tetracycline (25.00%), oxacillin (8.33%), vancomycin (0%) were significantly lower than the ones of erythromycin (100%), TMP/SMX (83.33%), clindamycin (91.67%), penicillin G(100%) (*p* < 0.001)

## Discussion

The causative pathogen of SSSS is the coagulase-positive group II *S.aureus* (especially strain 71). The skin acantholysis in the granular layer leads to the separation of the epidermis, flaccid skin bullae, and positive Nikolsky sign [12]. Children are the most susceptible population. The generalized SSSS is recommended to be admitted and treated with intravenous antibiotics due to the serious symptoms and the risk of occurrence of hypothermia, sepsis, and dehydration. Our study is one of the largest retrospective studies investigating the affecting factors of intravenous antibiotic treatment course in pediatric generalized SSSS patients. Consistent with the previous report by Handler MZ et al. [13], our study found that the male-to-female ratio was approximately equal (1.35:1) in children. The mean age of the patients was 2.47 ± 1.63 years old, 90.87% of patients were below 5 years old, which were also in line with the previous studies [13–16]. Most of the patients (68.04%) in our study came from the rural area. In the rural area, poor hygiene and medical support could be responsible for the transmission of the *S.aureus*, further research on the area difference of SSSS morbidity are needed. We found that the average maximum axillary temperature of the patients was 37.83 ± 0.78 °C, only 4.11% of patients presented with elevated CRP levels, the average WBC count was not very high. All of the characteristics above suggested that the generalized SSSS had a mild systemic stress response in the initial clinical course.

Although the diagnosis of SSSS is mainly based on clinical manifestation, it can be further confirmed by culturing *S. aureus* from any suspected primary focus of infection, such as the nasopharynx, periorificial, and diaper area [17]. Many studies pointed out that periorificial and nasopharyngeal cultures are the most likely to be positive, blood cultures are almost negative in children [5, 13, 18–21]. As confirmed by our data, higher positive culture rates were found in periorificial (54.55%) and throat swabs (30.77%) compared with blood samples (5.97%) in the pediatric population. Therefore, we consider that both periorificial and throat swabs maybe more useful than the blood samples for *S.aureus* culture. One reason may be that for most generalized SSSS patients, the bacteria only colonized on the skin and seldom entered the blood. However, generalized SSSS patients with complications such as sepsis were necessary to be included in the study and statistical analysis should be performed to draw an accurate conclusion on the difference of positive culture rates in different sample source.

Twenty four *S.aureus* strains were obtained and drug sensitivity tests demonstrated high resistant rates (91.67%) of clindamycin and low resistant rates of oxacillin (8.33%) and vancomycin (0). These results coincided with a previous report by Braunstein I et al. [22]. However, the clindamycin resistance rates in our study were significantly higher and the methicillin resistance rates were much lower compared to overall staphylococcal infections [14, 23]. The different frequencies of antibiotic application in our district may contribute to the different resistant profile of clindamycin and methicillin. Further research with large samples is needed to investigate the antibiotic-resistant profile of *S.aureus* in our city. In particular, two oxacillin-resistant *S.aureus* strains were successfully treated with intravenous cefazolin and external 2% fusidic acid cream rather than intravenous vancomycin in our study. It implied that the external application of 2% fusidic acid combined with intravenous antibiotic therapy played a role in eliminating the *S.aureus*. We hypothesized that the two *S.aureus* strains colonizing on the skin could be sensitive to fusidic acid, besides, the transepidermal absorption of the drug was favorable due to the application on the large body surface area and sound-absorbing capacity of skin in the pediatric patients. Liu Y et al. found the fusidic acid resistant rate among methicillin-resistant *S. aureus* (MRSA) was 0 according to a multicenter study of characterization of *S.aureus* from the skin and soft-tissue infections in China [24]. However, it should be noted that some resistant strains were emerging in some countries [25]. Further study is needed to investigate the fusidic acid sensitivity of oxacillin resistant *S.aureus* in our district.

In the final multiple linear regression model, younger ages were associated with longer intravenous antibiotic treatment duration. There have been reports demonstrating that lack of protective antibodies to exfoliative toxins, immature renal function with a decreased renal clearance of these toxins at a young age may account for the increased incidence at a younger age [13, 14, 26]. We speculate that older children could have increased titers of protective antibodies to exfoliative toxins and increased renal clearance of these toxins, which may be helpful for the reduction of the intravenous antibiotic treatment course.

Total WBC counts are used commonly to estimate the risk of serious bacterial infection. In SSSS, the leukocyte count can be elevated or normal [9]. There have been some studies to investigate the role of WBC count in evaluating the severity of infectious diseases. Some experts pointed out that absolute WBC and neutrophil count were not accurate enough for the identification of febrile children with potentially severe bacterial infection [27], Murray CK et.al found that bloodstream infection could not be reliably predicted by the indicators of WBC count [28]. Peltola V et.al stated that an elevated WBC count be detected in only one fifth child with *S.aureus* infection in bloodstream [29]. These results above were contradictory to our ones.. Schrock et al. found in patients with skin and soft tissue infections, the elevated WBC were significantly associated with prolonged length of hospital stay in patients with skin and soft tissue infections [30]. The conclusion above was consistent with our one that the higher level of total WBC count can indicate longer intravenous antibiotic treatment course of generalized SSSS in pediatric patients without systematic infection.

We found that compared with the patients with normal CRP values, the patients with elevated ones had a longer intravenous antibiotic treatment course (β = 1.64, [95%CI: 0.49, 2.78], *p* < 0.01). It has been confirmed that the CRP levels increased during bacterial infection and thus CRP was considered as a marker for inflammation [31]. Some researchers revealed that there was a remarkable increasing of mean CRP level in the patients with chronic *S.aureus* infection compared with the healthy population [32, 33]. Furthermore, CRP was considered to be useful to assess the severity of some bacterial infections [34, 35]. Based on the evidence of all these studies above, we speculate that CRP level can assist with the evaluation of the severity of generalized SSSS, therefore patients with elevated CRP level needed more days of intravenous antibiotic application than the ones with normal CRP level.

In our study, the external application of fusidic acid other than mupirocin helped to reduce the intravenous antibiotic treatment course. The reason may be that the fusidic acid were favorable in eliminating the colonization of *S.aureus*, which has been confirmed by some previous studies [36, 37]. What is noteworthy is that the resistant rates of MRSA strains to fusidic acid were low [38]. Liu Y et al. discovered that topical application of fusidic acid was effective for the treatment of skin and soft-tissue infections caused by *S.aureus* [24]. Of note, in our study, we found fusidic acid had a more obvious effect than mupirocin on reducing the days of intravenous antibiotic application. We speculated the reason might be the lower resistance rate of *S.aureus* to fusidic acid than to mupirocin in China. Some Chinese experts have already compared the resistance rate of mupirocin with the one of fusidic acid. Liu YC et.al observed that only 1.7% of the methicillin-sensitive *S. aureus* strains were resistant to fusidic acid and 3.0% of the MRSA strains were resistant to fusidic acid [39]**.** Liu QZ et al. performed an observational study and found that among the 53 high-level mupirocin-resistant MRSA strains, none was resistant to fusidic acid [40]. Similar results were exhibited by Liu X et al. [41], they found that the resistance rate of fusidic acid (2.9%) was much lower than mupirocin (17.6%). According to our study, most of the patients were topically administered with mupirocin rather than fusidic acid, frequent application of mupirocin may contribute to the induction of the drug resistance in our district. Future prospective studies can be focused on examining the effects of different topical antibiotic in decreasing need for intravenous therapy.

There were some limitations in our study. Firstly, we did not have data on phage types of the isolated *S. aureus* and toxins responsible for SSSS to further confirm the culprit pathogen and diagnosis. Secondly, we did not perform the antimicrobial sensitivity tests of fusidic acid and mupirocin among the *S.aureus* strains. Multicenter prospective research with further laboratory investigations of *S.aureus* is necessary for the future.

## Conclusions

It can be concluded that younger age, elevated WBC and CRP levels indicated longer length of intravenous antibiotic application, external application of fusidic acid other than mupirocin helped to reduce the intravenous antibiotic treatment course. Compared with blood samples, the culture positive rates of *S.aureus* in periorificial and throat swabs were higher. Clindamycin-resistant isolates were much more common than oxacillin-resistant and vancomycin-resistant ones, suggesting clindamycin monotherapy for SSSS should be avoided. Oxacillin resistance was rare and amoxicillin-clavulanic acid, cefazolin, and cefuroxime were still effective in treating SSSS.

## Data Availability

The data that support the findings of this study are available from the corresponding author upon reasonable request.

## Abbreviations

SSSS: Staphylococcal Scalded Skin Syndrome
*Staphylococcus aureus*: *S. aureus*
CRP: C-reactive protein
TMP/SMX: Trimethoprim-sulfamethoxazole
ETA: Exfoliative toxin A
ETB: Exfoliative toxin B
ICD-10: International Classification of Diseases, tenth revision
WBC: White blood cell
SD: Standard deviations
IQR: Interquartile range
MRSA: Methicillin-resistant *S. aureus*
T_max_: Maximum axillary temperature
IV: Intravenous

## References

[1] Kapoor V, Travadi J, Braye S (2008). Staphylococcal scalded skin syndrome in an extremely premature neonate: a case report with a brief review of literature. J Paediatr Child Health.

[2] Ladhani S, Joannou CL, Lochrie DP, Evans RW, Poston SM (1999). Clinical, microbial, and biochemical aspects of the exfoliative toxins causing staphylococcal scalded-skin syndrome. Clin Microbiol Rev.

[3] Ladhani S (2003). Understanding the mechanism of action of the exfoliative toxins of Staphylococcus aureus. FEMS Immunol Med Microbiol.

[4] Ladhani S (2001). Recent developments in staphylococcal scalded skin syndrome. Clin Microbiol Infect.

[5] Lamand V, Dauwalder O, Tristan A, Casalegno JS, Meugnier H, Bes M, Dumitrescu O, Croze M, Vandenesch F, Etienne J, Lina G (2012). Epidemiological data of staphylococcal scalded skin syndrome in France from 1997 to 2007 and microbiological characteristics of Staphylococcus aureus associated strains. Clin Microbiol Infect.

[6] Stainman A, Hsu DY, Silerberg JI (2018). Epidemiology of staphylococcal scalded skin syndrome in US children. Br J Dermatol.

[7] Arnold JD, Hoek SN, Kirkorian AY (2018). Epidemiology of staphylococcal scalded skin syndrome in the United States: a cross-sectional study, 2010-2014. J Am Acad Dermatol.

[8] King RW, Carone HL, Victor PS (2019). Staphylococcal Scalded Skin Syndrome (SSSS).

[9] Bolognia JL, Jorizzo JL, Schaffer JV, Millett CR, Halpern AV, Reboli AC, Heymann WR (2012). Dermatology. Bacterial diseases.

[10] Leung A, Barankin B, Leong KF (2018). Staphylococcal-scalded skin syndrome: evaluation, diagnosis, and management. World J Pediatr.

[11] Goldstein B, Giroir B, Randolph A, International Consensus Conference on Pediatric Sepsis (2005). International pediatric sepsis consensus conference: definitions for sepsis and organ dysfunction in pediatrics. Pediatr Crit Care Med.

[12] Nishifuji K, Shimizu A, Ishiko A, Iwasaki T (2010). Removal of amino-terminal extracellular domains of desmoglein 1 by staphylococcal exfoliative toxin is sufficient to initiate epidermal blister formation. J Dermatol Sci.

[13] Handler MZ, Schwartz RA (2014). Staphylococcal scalded skin syndrome: diagnosis and management in children and adults. J Eur Acad Dermatol Venereol.

[14] Mishra AK, Yadav P, Mishra A (2016). A systemic review on staphylococcal scalded skin syndrome (SSSS): a rare and critical disease of neonates. Open Microbiol J.

[15] Oliveira AR, Aires S, Faria C (2013). Staphylococcal scalded skin syndrome. BMJ Case Rep.

[16] Patel GK, Finlay AY (2003). Staphylococcal scalded skin syndrome: diagnosis and management. Am J Clin Dermatol.

[17] Davidson J, Polly S, Hayes PJ, Fisher K, Talati A, Patel T (2017). Recurrent staphylococcal scalded skin syndrome in an extremely low-birth-weight neonate. AJP Rep.

[18] Li MY, Hua Y, Wei GH, Qiu L (2014). Staphylococcal scalded skin syndrome in neonates: an 8-year retrospective study in a single institution. Pediatr Dermatol.

[19] Lipový B, Brychta P, Chaloupková Z, Suchánek I (2012). Staphylococcal scalded skin syndrome in the Czech Republic: an epidemiological study. Burns..

[20] Paranthaman K, Bentley A, Milne LM, et al. Nosocomial outbreak of staphyloccocal scalded skin syndrome in neonates in England, December 2012 to March 2013. Euro Surveill. 2014;19(33). 10.2807/1560-7917.es2014.19.33.20880. 10.2807/1560-7917.es2014.19.33.20880 25166346

[21] Wang Z, Feig JL, Mannschreck DB, Cohen BA (2020). Antibiotic sensitivity and clinical outcomes in staphylococcal scalded skin syndrome. Pediatr Dermatol.

[22] Braunstein I, Wanat KA, Abuabara K, McGowan KL, Yan AC, Treat JR (2014). Antibiotic sensitivity and resistance patterns in pediatric staphylococcal scalded skin syndrome. Pediatr Dermatol.

[23] Hodille E, Rose W, Diep BA, Goutelle S, Lina G, Dumitrescu O (2017). The role of antibiotics in modulating virulence in Staphylococcus aureus. Clin Microbiol Rev.

[24] Liu Y, Xu Z, Yang Z, Sun J, Ma L (2016). Characterization of community-associated Staphylococcus aureus from skin and soft-tissue infections: a multicenter study in China. Emerg Microbes Infect.

[25] Doudoulakakis A, Spiliopoulou I, Spyridis N, Giormezis N, Kopsidas J, Militsopoulou M, Lebessi E, Tsolia M (2017). Emergence of a Staphylococcus aureus clone resistant to Mupirocin and Fusidic acid carrying exotoxin genes and causing mainly skin infections. J Clin Microbiol.

[26] Jeyakumari D, Gopal R, Eswaran M, MaheshKumar C (2009). Staphylococcal scalded skin syndrome in a newborn. J Glob Infect Dis.

[27] De S, Williams GJ, Hayen A (2014). Value of white cell count in predicting serious bacterial infection in febrile children under 5 years of age. Arch Dis Child.

[28] Murray CK, Hoffmaster RM, Schmit DR, Hospenthal DR, Ward JA, Cancio LC, Wolf SE (2007). Evaluation of white blood cell count, neutrophil percentage, and elevated temperature as predictors of bloodstream infection in burn patients. Arch Surg.

[29] Peltola V, Mertsola J, Ruuskanen O (2006). Comparison of total white blood cell count and serum C-reactive protein levels in confirmed bacterial and viral infections. J Pediatr.

[30] Schrock JW, Laskey S, Cydulka RK (2008). Predicting observation unit treatment failures in patients with skin and soft tissue infections. Int J Emerg Med.

[31] Healy B, Freedman A (2006). Infections. BMJ.

[32] Povoa P, Coelho L, Almeida E (2005). C-reactive protein as a marker of infection in critically ill patients. Clin Microbiol Infect.

[33] Sproston NR, Ashworth JJ (2018). Role of C-reactive protein at sites of inflammation and infection. Front Immunol.

[34] Almirall J, Bolíbar I, Toran P, Pera G, Boquet X, Balanzó X, Sauca G, Community-Acquired Pneumonia Maresme Study Group (2004). Contribution of C-reactive protein to the diagnosis and assessment of severity of community-acquired pneumonia. Chest..

[35] Hohenthal U, Hurme S, Helenius H, Heiro M, Meurman O, Nikoskelainen J, Kotilainen P (2009). Utility of C-reactive protein in assessing the disease severity and complications of community-acquired pneumonia. Clin Microbiol Infect.

[36] Pimentel de Araujo F, Tinelli M, Battisti A, Ercoli A, Anesi A, Pantosti A, Monaco M (2018). An outbreak of skin infections in neonates due to a Staphylococcus aureus strain producing the exfoliative toxin A. Infection..

[37] Meshram GG, Kaur N, Hura KS (2018). Staphylococcal scalded skin syndrome: A pediatric dermatology case report. SAGE Open Med Case Rep.

[38] Chen W, He C, Yang H, Shu W, Cui Z, Tang R, Zhang C, Liu Q (2020). Prevalence and molecular characterization of methicillin-resistant Staphylococcus aureus with mupirocin, fusidic acid and/or retapamulin resistance. BMC Microbiol.

[39] Liu YC, Gengg WJ, Yang YH (2012). Susceptibility to and resistance determinants of fusidic acid in Staphylococcus aureus isolated from Chinese children with skin and soft tissue infections. FEMS Immunol Med Microbiol.

[40] Liu QZ, Wu Q, Zhang YB, Liu MN, Hu FP, Xu XG, Zhu DM, Ni YX (2010). Prevalence of clinical meticillin-resistant Staphylococcus aureus (MRSA) with high-level mupirocin resistance in Shanghai and Wenzhou, China. Int J Antimicrob Agents.

[41] Liu X, Deng S, Huang J, Huang Y, Zhang Y, Yan Q, Wang Y, Li Y, Sun C, Jia X (2017). Dissemination of macrolides, fusidic acid and mupirocin resistance among Staphylococcus aureus clinical isolates. Oncotarget..

